# Supplementary material for: “Satanism is witchcraft’s younger sibling”: Changing perceptions of natural and supernatural anaemia causality in Malawian children
Source: PLoS One. 2021 Apr 30;16(4):e0250661. doi: 10.1371/journal.pone.0250661 (PMC8087048; doi:10.1371/journal.pone.0250661)
Supplement: Attachment — Submitted filename: Response to Reviewers.pdf [file pone.0250661.s001.pdf]

Response to reviewers

We would like to thank academic editor Danielle Poole and the two reviewers for evaluating our manuscript titled "*Satanism is witchcraft's younger sibling": Changing perceptions of natural and supernatural anaemia causality in Malawian children*. We greatly appreciate the constructive comments provided by the reviewers and believe the changes have improved the manuscript.

Response to comments from reviewer 1

1. *The authors conclude that caregivers may associate the child's anaemia symptoms with witchcraft, satanism or a local illness (such as kakozi and kapamba). However, participants were asked about these specific topics. It may have been possible that other causes would have come up if the questions would not have been guided to these causes. This may be added as a limitation of the study and/or the conclusion may be better formulated that 'when' caregivers associate child's anaemia symptoms with witchcraft, satanism or a local illness ..... traditional treatment may be sought initially which may lead to delayed hospital care of sick children.*

As suggested by reviewer 1, the wording in the conclusion is now changed to "When caregivers relate the child's anaemia symptoms to these causes it is likely that traditional and complementary treatment at household or community level is sought initially" (pg 28). The following sentence has been added to the section on study limitations; "Participants were asked about specific topics guided by previous fieldwork and it is possible that other causes of anaemia would have come up if questions were not directed to these specific topics" (pg 29).

2*. Suggest to take out parts on sickle cell anaemia – as it does not add anything of interest.*

We agree that there is no need to mention sickle cell anaemia since the collected data on this topic was sparse, and therefore we have removed the following sentence in section 2.4 Data analysis; "Results from all topics in the guide except sickle cell anaemia will be presented. The reason is that very few of the participants knew of this condition" (pg 8). Although no results regarding this topic is presented in the results section, we still feel that it should be listed as one of the seven topics in the FGD guide in section 2.2 Data collection (pg 7).

***3.** The inclusion criteria for participants are not clear. Why needed the parents to be preferably in the age group 18-35 and was this defined as 'young'? When reporting about young mothers or young fathers - in which age group were they?*

To clarify the inclusion criteria, the sentence in section 2.3 on parents being "preferably within the age range 18-35 years" has been taken out (pg 7). Throughout the manuscript, the wording is now changed from 'young mothers' and 'young fathers' to simply 'mothers' and 'fathers', and from 'young parents' to 'parents'. We do not specifically mention the ages of the parents across the various FGDs, but the age range of the sample as a whole is given in section 3.1 (pg 9).

***4.** Discussion: I would suggest to start with a paragraph that summarizes the results.*

The first paragraph of the discussion now describes the main findings of the study. We did not add any new sentences, but removed the first part of the discussion since it did not summarise the results. Parts of the original discussion section is now added to the introduction where it seems to fit better (pg 3).

***5.** Conclusion: the 'hindering of timely hospital care such as iron supplements and blood transfusion' seems out of place in the Conclusion section – as this is not discussed in the Discussion section.*
The above-mentioned sentence is removed since there is, as highlighted by reviewer 1, limited focus on hospital treatment of anaemia elsewhere in the manuscript.

Response to comments from reviewer 2

***1**. Although data on the characteristics and socioeconomic status of the participants were given, these were not discussed further in the context of the findings from the focus group discussions. a. For example, it would have been interesting to know if there were any differences in beliefs on Satanism in the Moslem participants, given that the paper said that belief in Satanism was directly linked to the Christian faith.*

a. Thank you for highlighting the point about Muslim participants and their views on Satanism. Out of the three Muslim participants only one, a father in FGD 5, spoke explicitly about

Satanism and described it as a witchcraft-related, magical practice where people are killed mysteriously. We wish that we had more data to compare the views on Satanism among Muslims and Christians. Due to the limited number of Muslim participants we consider the data on this topic to be too narrow for drawing any conclusions on whether there is a difference across the two religions.

b. Also, were there any differences in health seeking behaviour based on the level of final education of the caregivers?
If the numbers were too small to detect any such differences, it would be worth mentioning, and/or a comment from the authors as to whether religion and/or education (or any other SES factors) may play any role in the decision-making tree of the caregivers.

b. As suggested by reviewer 2 we have mentioned how final level of formal education seemed to influence health-seeking behaviour among participants in this study. In section 3.2 the following paragraph has been included; "Treatment-seeking behaviour and decision-making processes were related to reported level of formal education. Participants with secondary education (grades 9-12), of whom were mostly fathers, would emphasise the importance of accessing hospital care as a primary strategy. They spoke avidly about the 'professionalism' and 'skills' of medical doctors and health personnel, and many of them would only resort to traditional medicine if hospital medicine failed. On the contrary, those with limited or no formal education, of whom were mainly grandfathers and grandmothers, strongly advocated the use of home-based herbal remedies. They also explained how they would initially seek help and guidance from traditional healers and community elders prior to accessing hospital as a secondary strategy" (pg 12-13).

While looking into another SES factor, namely area of residence, we did not find any clear differences in decision-making and health-seeking behaviour between groups situated in rural versus semi-rural villages.

2. *One assumes that the participants were all independent, i.e., they were parents or grandparents of 90 separate children? This should be explicitly stated, and if this was not the case, it should be clear how many participants shared the same child and a statement made about how this might bias the results.*

In section 2.3 the following sentence is added to clarify the participant selection; “None of the participants were from the same household” (pg 7). Thus, this study included 90 participants from 90 separate homes.
